# Identification of genetically driven Therapeutic Targets for Alzheimer’s disease

**DOI:** 10.1002/alz.093138

**Published:** 2025-01-03

**Authors:** Alison M. Goate, Edoardo Marcora, Carlos Cruchaga

**Affiliations:** ^1^ Ronald M. Loeb Center for Alzheimer’s Disease, Friedman Brain Institute, Icahn School of Medicine at Mount Sinai, New York, NY USA; ^2^ Washington University in St. Louis, School of Medicine, St. Louis, MO USA

## Abstract

Therapeutics against targets supported by human genetics are more than twice as likely to make it to the clinic as an FDA approved drug. In 2012 the National Institute on Aging in response to the U.S. congressional initiative established the Alzheimer’s Disease Sequencing Project (ADSP), a large‐scale genetics effort to further our fundamental understanding of Alzheimer disease and related disorders (ADRD) with the goal to accelerate the development of effective treatments for ADRD. The ADSP has used genome‐wide association studies (GWAS) of SNP array data and whole genome sequencing to identify more than one hundred loci associated with Alzheimer’s disease (AD) risk. Post GWAS analyses has identified candidate causal genes for a good proportion of these loci. We integrated human genetics and functional genomics to identify the cell types, genes and pathways that may modulate AD risk. Induced pluripotent stem cells and multi‐OMICs were used to validate the impact of risk alleles and genes on AD relevant phenotypes in vitro and in vivo. Functional genomics has implicated myeloid cells including microglia and other macrophages in AD risk. Pathway‐based analyses demonstrate that efferocytosis is a disease risk hub and that TREM2 signaling is implicated by multiple risk genes. Immunotherapies targeting TREM2, MS4A4A, MS4A6A are under development. Progress toward developing therapies to these and other targets will be reviewed. Immunotherapies, gene‐based and small molecule approaches are being developed to target TREM2 signaling, APOE biology and other aspects of macrophage/microglial function such as efferocytosis.

